# Bis(*N*-*sec*-butyl-*N*-*n-*propyl­dithio­carbamato-κ^2^ *S*,*S*′)(1,10-phenanthroline-κ^2^ *N*,*N*′)zinc(II)

**DOI:** 10.1107/S1600536810033672

**Published:** 2010-08-28

**Authors:** Amna Salem Alzaalouk, Ibrahim Baba, Mohamed Ibrahim Mohamed Tahir, Seik Weng Ng, Edward R. T. Tiekink

**Affiliations:** a School of Chemical Sciences and Food Technology, Faculty of Science and Technology, Universiti Kebangbaan Malaysia, 43600 Bangi, Malaysia; b Department of Chemistry, Universiti Putra Malaysia, 43400 Serdang, Malaysia; c Department of Chemistry, University of Malaya, 50603 Kuala Lumpur, Malaysia

## Abstract

Two independent but very similar mol­ecules comprise the asymmetric unit of the title compound, [Zn(C_8_H_16_NS_2_)_2_(C_12_H_8_N_2_)]. The N_2_S_4_ donor set about Zn is defined by two symmetrically chelating dithio­carbamate ligands and a 1,10-phenanthroline ligand. Distortions from the ideal octa­hedral coordination geometry arise from the restricted bite angles of the ligands. The main feature of the crystal packing is the formation of tetra­meric supra­molecular aggregates mediated by C—H⋯S inter­actions. Disorder was found in each of the *sec*-butyl groups. This was resolved over two positions in each case with the major components of the disorder having site occupancies in the range 0.551 (6)–0.725 (5).

## Related literature

For a review on the supra­molecular aggregation patterns of zinc-triad dithio­carbamates, see: Tiekink (2003 ▶). For crystal engineering studies on dithio­carbamates and their zinc compounds, see: Benson *et al.* (2007 ▶); Howie *et al.* (2008 ▶). For the structure of the mononuclear binary precursor compound, see: Awang *et al.* (2010 ▶).
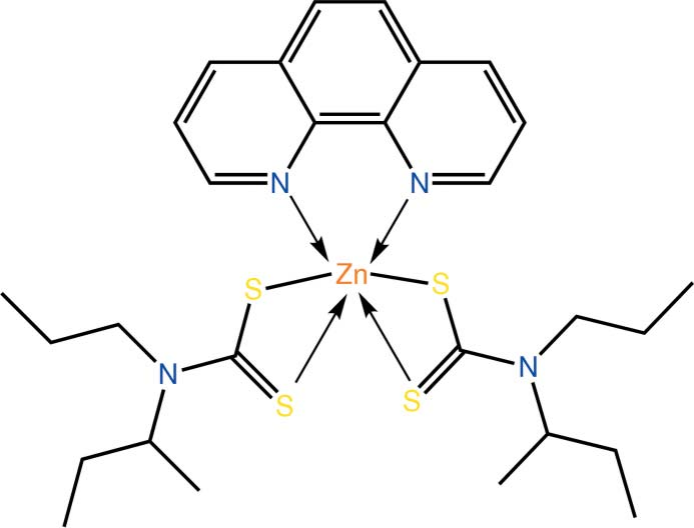


## Experimental

### 

#### Crystal data

[Zn(C_8_H_16_NS_2_)_2_(C_12_H_8_N_2_)]
*M* *_r_* = 626.25
Triclinic, 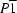

*a* = 13.4195 (5) Å
*b* = 14.3946 (5) Å
*c* = 16.6444 (5) Å
α = 92.727 (3)°
β = 90.998 (3)°
γ = 103.368 (3)°
*V* = 3123.18 (18) Å^3^
*Z* = 4
Mo *K*α radiation
μ = 1.08 mm^−1^
*T* = 100 K
0.30 × 0.25 × 0.10 mm

#### Data collection

Oxford Diffraction Xcaliber Eos Gemini diffractometer
Absorption correction: multi-scan (*CrysAlis PRO*; Oxford Diffraction, 2010 ▶) *T*_min_ = 0.824, *T*_max_ = 1.000
53206 measured reflections
14179 independent reflections
10260 reflections with *I* > 2σ(*I*)
*R*_int_ = 0.034

#### Refinement

*R*[*F* ^2^ > 2σ(*F* ^2^)] = 0.034
*wR*(*F* ^2^) = 0.099
*S* = 1.05
14179 reflections
790 parameters
302 restraints
H-atom parameters constrained
Δρ_max_ = 1.32 e Å^−3^
Δρ_min_ = −0.59 e Å^−3^

### 

Data collection: *CrysAlis PRO* (Oxford Diffraction, 2010 ▶); cell refinement: *CrysAlis PRO*; data reduction: *CrysAlis PRO*; program(s) used to solve structure: *SHELXS97* (Sheldrick, 2008 ▶); program(s) used to refine structure: *SHELXL97* (Sheldrick, 2008 ▶); molecular graphics: *ORTEP-3* (Farrugia, 1997 ▶) and *DIAMOND* (Brandenburg, 2006 ▶); software used to prepare material for publication: *publCIF* (Westrip, 2010 ▶).

## Supplementary Material

Crystal structure: contains datablocks global, I. DOI: 10.1107/S1600536810033672/bt5329sup1.cif

Structure factors: contains datablocks I. DOI: 10.1107/S1600536810033672/bt5329Isup2.hkl

Additional supplementary materials:  crystallographic information; 3D view; checkCIF report

## Figures and Tables

**Table 1:** Hydrogen-bond geometry (Å, °)

*D*—H⋯*A*	*D*—H	H⋯*A*	*D*⋯*A*	*D*—H⋯*A*
C28—H28a⋯S8^i^	0.95	2.80	3.560 (2)	137
C50—H50⋯S8^ii^	0.95	2.80	3.702 (3)	158

Symmetry codes: (i) 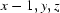; (ii) 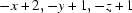.

**Fig. 1.**
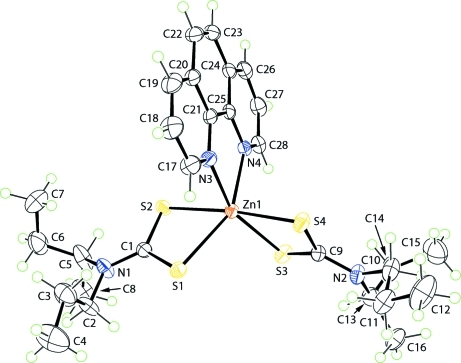
The molecular structure of the first independent molecule in (I) showing the atom-labelling scheme and displacement ellipsoids at the 50% probability level. Only the major components of the disordered atoms associated with the sec-butyl groups are shown for reasons of clarity.

**Fig. 2.**
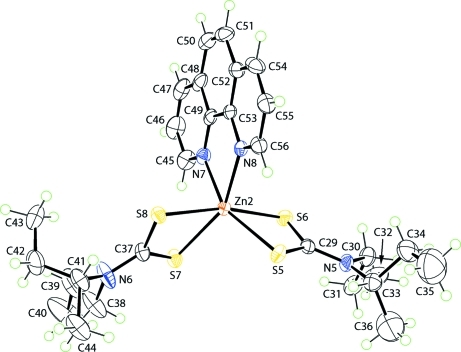
The molecular structure of the second independent molecule in (I) showing the atom-labelling scheme and displacement ellipsoids at the 50% probability level. Only the major components of the disordered atoms associated with the sec-butyl groups are shown for reasons of clarity.

**Fig. 3.**
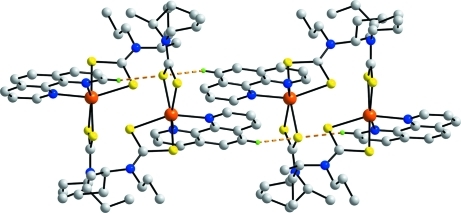
Supramolecular tetrameric unit in (I) mediated by C–H···S interactions, shown as orange dashed lines. Hydrogen atoms not involved in the C–H···S interactions have been omitted for reasons of clarity.

**Fig. 4.**
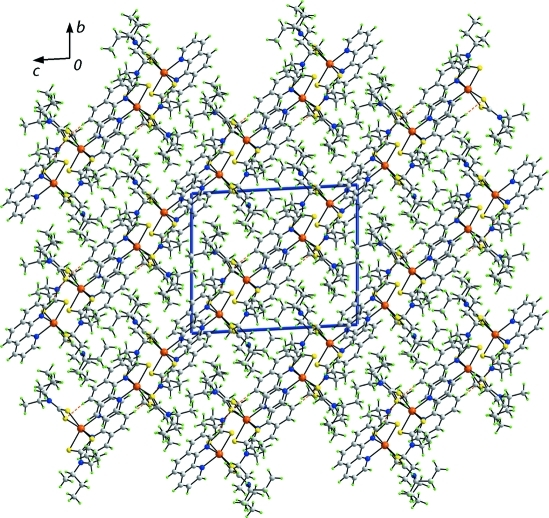
A view in projection down the a axis showing the inter-digitation of layers in (I).

## supplementary crystallographic information

### Comment

Dithiocarbamate anions (Howie *et al.*, 2008) and their zinc derivatives, including their adducts with nitrogen-containing bases (Benson *et al.*, 2007), attract attention in crystal engineering endeavours. Often the polynuclear structures found in the structures of the parent binary precursor compounds are broken down in the presence of base. However, in the present study, the parent binary compound Zn[S_2_N(*sec*-Bu)(*n*-Pr)]_2_ (Awang *et al.*, 2010) was found to be mononuclear in its crystal structure, an observation consistent with the influence of the relatively large *iso*-butyl groups (Tiekink, 2003). Not surprisingly, its 1:1 1,10-phenanthroline adduct, (I), is also mononuclear.

The crystallographic asymmetric unit of (I) comprises two very similar molecules, Figs 1 and 2. Each Zn atom is coordinated by two dithiocarbamate ligands which form similar Zn–S bond distances, Table 1, with the range being relatively narrow, *i.e*. 2.4881 (6) Å, for Zn2–S6, to 2.5311 (6) Å, for Zn1–S2. Similarly, the Zn–N bond distances span a narrow range, Table 1. The coordination geometry is based on an octahedron with deviations related to the restricted bite distances of the chelating ligands.

The presence of C–H···S interactions, Table 1, lead to the formation of tetrameric supramolecular aggregates in the crystal structure of (I). The S8 atom is pivotal in these. The molecules comprising the asymmetric unit are connected by the C–H···S8 interaction involving the C28–H28*a* atom. The dimeric aggregates thus formed are linked *via* the C–H···S8 interaction involving the C50–H50 atom, Fig. 3. Globally, the molecules pack into layers parallel to (0 1 1) and inter-digitate, Fig. 4.

### Experimental

Carbon disulfide (20 mmol) was dropped into an ethanol solution (100 ml) of *N*-*sec*-butyl-*N*-*n*-propylamine (20 mmol). The solution was kept at 273 K for an hour. Zinc chloride (10 mmol) dissolved in ethanol (50 ml) was added to give a white precipitate. This was collected and redissolved in chloroform (50 ml). The solution was mixed with a solution of 1,10-phenanthroline (10 mmol) dissolved in ethanol (10 mol). The yellow solution was set aside for the growth of crystals.

### Refinement

H-atoms were placed in calculated positions (C—H 0.95 to 1.00 Å) and were included in the refinement in the riding model approximation, with *U*_iso_(H) set to 1.2 to 1.5*U*_equiv_(C).

All *sec*-butyl groups were found to be disordered. For each group, the site occupancies were refined.  The major component  had site occupancy factor  of 0.551 (6) for the C5-containing group, 0.725 (5) for the C13-group, 0.587 (6) for the C33-group, and 0.557 (6) for the C41-group. For both the ordered *n*-propyl and disordered *sec*-butyl groups, the 1,2-related C–C distances were tightly restrained to 1.500±0.005 Å and the 1,3-related ones to 2.51±0.01 Å. Within the S_2_C–NC_2_ fragment, the S_2_C–N distances were restrained to 1.35±0.01 Å and the *N*–C_alkyl_ distances to 1.45±0.01 Å. Finally, all anisotropic displacement parameters were refined individually except for the C33' and C36' atoms which were refined isotropically. Additionally, the anisotropic displacement parameters for the carbon atoms of both the ordered *n*-propyl and disordered *sec*-butyl groups were tightly restrained to be nearly isotropic.

The maximum and minimum residual electron density peaks of 1.32 and 0.59 eÅ^-3^, respectively, were located 0.50 Å and 0.28 Å from the H36*b* and C40 atoms, respectively.

### Crystal data

**Table:**

[Zn(C_8_H_16_NS_2_)_2_(C_12_H_8_N_2_)]	*Z* = 4
*M**_r_* = 626.25	*F*(000) = 1320
Triclinic, *P*1	*D*_x_ = 1.332 Mg m^−^^3^
Hall symbol: -P 1	Mo *K*α radiation, λ = 0.71073 Å
*a* = 13.4195 (5) Å	Cell parameters from 25754 reflections
*b* = 14.3946 (5) Å	θ = 2.2–29.2°
*c* = 16.6444 (5) Å	µ = 1.08 mm^−^^1^
α = 92.727 (3)°	*T* = 100 K
β = 90.998 (3)°	Prism, yellow
γ = 103.368 (3)°	0.30 × 0.25 × 0.10 mm
*V* = 3123.18 (18) Å^3^	

### Data collection

**Table:**

Oxford Diffraction Xcaliber Eos Gemini diffractometer	14179 independent reflections
Radiation source: fine-focus sealed tube	10260 reflections with *I* > 2σ(*I*)
graphite	*R*_int_ = 0.034
Detector resolution: 16.1952 pixels mm^-1^	θ_max_ = 27.5°, θ_min_ = 2.2°
ω scans	*h* = −17→17
Absorption correction: multi-scan (*CrysAlis PRO*; Oxford Diffraction, 2010)	*k* = −18→18
*T*_min_ = 0.824, *T*_max_ = 1.000	*l* = −21→21
53206 measured reflections	

### Refinement

**Table:**

Refinement on *F*^2^	Primary atom site location: structure-invariant direct methods
Least-squares matrix: full	Secondary atom site location: difference Fourier map
*R*[*F*^2^ > 2σ(*F*^2^)] = 0.034	Hydrogen site location: inferred from neighbouring sites
*wR*(*F*^2^) = 0.099	H-atom parameters constrained
*S* = 1.05	*w* = 1/[σ^2^(*F*_o_^2^) + (0.0566*P*)^2^] where *P* = (*F*_o_^2^ + 2*F*_c_^2^)/3
14179 reflections	(Δ/σ)_max_ = 0.001
790 parameters	Δρ_max_ = 1.32 e Å^−^^3^
302 restraints	Δρ_min_ = −0.59 e Å^−^^3^

### Fractional atomic coordinates and isotropic or equivalent isotropic displacement parameters (Å^2^)

**Table:**

	*x*	*y*	*z*	*U*_iso_*/*U*_eq_	Occ. (<1)
Zn1	0.239825 (18)	0.874711 (17)	0.166771 (14)	0.02154 (7)	
Zn2	0.801467 (18)	0.639792 (17)	0.331410 (14)	0.02174 (7)	
S1	0.35079 (4)	1.00240 (4)	0.25789 (3)	0.02757 (13)	
S2	0.12697 (4)	0.96605 (4)	0.24166 (3)	0.02515 (13)	
S3	0.23307 (4)	0.72779 (4)	0.24177 (3)	0.02953 (14)	
S4	0.36271 (5)	0.78842 (4)	0.10466 (3)	0.02983 (14)	
S5	0.79867 (4)	0.78922 (4)	0.26012 (3)	0.02810 (13)	
S6	0.67551 (4)	0.71816 (4)	0.39812 (3)	0.02944 (13)	
S7	0.68307 (4)	0.50772 (4)	0.25064 (4)	0.02980 (14)	
S8	0.90639 (4)	0.56207 (4)	0.23704 (3)	0.02479 (13)	
N1	0.23999 (13)	1.10333 (14)	0.34135 (11)	0.0317 (5)	
N2	0.37212 (14)	0.63516 (13)	0.18675 (11)	0.0293 (4)	
N3	0.25422 (13)	0.96448 (13)	0.06232 (10)	0.0242 (4)	
N4	0.09827 (13)	0.81818 (12)	0.09271 (10)	0.0218 (4)	
N5	0.66886 (13)	0.88235 (13)	0.33127 (11)	0.0289 (4)	
N6	0.78352 (14)	0.42815 (15)	0.13972 (13)	0.0452 (6)	
N7	0.80350 (14)	0.55248 (13)	0.43810 (11)	0.0260 (4)	
N8	0.94593 (13)	0.70603 (12)	0.39861 (10)	0.0224 (4)	
C1	0.23924 (16)	1.03239 (14)	0.28596 (12)	0.0224 (5)	
C2	0.33750 (16)	1.15812 (17)	0.37841 (13)	0.0342 (6)	
H2A	0.3815	1.1133	0.3893	0.041*	
H2B	0.3233	1.1872	0.4308	0.041*	
C3	0.39618 (19)	1.23626 (19)	0.32837 (15)	0.0455 (7)	
H3A	0.4049	1.2091	0.2739	0.055*	
H3B	0.3565	1.2858	0.3227	0.055*	
C4	0.5000 (2)	1.2816 (2)	0.36624 (19)	0.0721 (10)	
H4A	0.5362	1.3319	0.3325	0.108*	
H4B	0.5398	1.2329	0.3711	0.108*	
H4C	0.4915	1.3096	0.4198	0.108*	
C5	0.1437 (4)	1.1151 (4)	0.3785 (4)	0.045 (2)	0.551 (6)
H5	0.0854	1.0719	0.3473	0.054*	0.551 (6)
C6	0.1371 (5)	1.2168 (4)	0.3660 (3)	0.0583 (19)	0.551 (6)
H6A	0.1984	1.2607	0.3917	0.070*	0.551 (6)
H6B	0.0763	1.2288	0.3936	0.070*	0.551 (6)
C7	0.1300 (10)	1.2400 (7)	0.2786 (4)	0.077 (3)	0.551 (6)
H7A	0.1256	1.3068	0.2755	0.115*	0.551 (6)
H7B	0.0688	1.1979	0.2528	0.115*	0.551 (6)
H7C	0.1911	1.2304	0.2511	0.115*	0.551 (6)
C8	0.1342 (9)	1.0898 (6)	0.4651 (4)	0.057 (3)	0.551 (6)
H8A	0.1391	1.0233	0.4696	0.086*	0.551 (6)
H8B	0.0679	1.0972	0.4846	0.086*	0.551 (6)
H8C	0.1895	1.1322	0.4975	0.086*	0.551 (6)
C5'	0.1483 (5)	1.1437 (5)	0.3539 (4)	0.035 (2)	0.449 (6)
H5'	0.0902	1.0922	0.3309	0.041*	0.449 (6)
C6'	0.1248 (5)	1.1511 (4)	0.4421 (3)	0.0372 (17)	0.449 (6)
H6'A	0.1728	1.2079	0.4679	0.045*	0.449 (6)
H6'B	0.0546	1.1610	0.4472	0.045*	0.449 (6)
C7'	0.1329 (12)	1.0645 (7)	0.4866 (6)	0.047 (2)	0.449 (6)
H7'A	0.1155	1.0734	0.5429	0.071*	0.449 (6)
H7'B	0.2030	1.0558	0.4840	0.071*	0.449 (6)
H7'C	0.0853	1.0079	0.4618	0.071*	0.449 (6)
C8'	0.1378 (10)	1.2314 (7)	0.3107 (5)	0.062 (3)	0.449 (6)
H8'A	0.1546	1.2239	0.2541	0.094*	0.449 (6)
H8'B	0.1847	1.2881	0.3359	0.094*	0.449 (6)
H8'C	0.0672	1.2388	0.3142	0.094*	0.449 (6)
C9	0.32818 (16)	0.70920 (15)	0.17887 (13)	0.0245 (5)	
C10	0.44458 (17)	0.61559 (17)	0.12801 (15)	0.0392 (6)	
H10A	0.4203	0.6278	0.0739	0.047*	
H10B	0.4451	0.5469	0.1282	0.047*	
C11	0.55093 (17)	0.67274 (19)	0.14187 (16)	0.0450 (7)	
H11A	0.5780	0.6578	0.1942	0.054*	
H11B	0.5513	0.7417	0.1440	0.054*	
C12	0.6207 (2)	0.6506 (2)	0.07366 (19)	0.0656 (10)	
H12A	0.6905	0.6891	0.0838	0.098*	
H12B	0.5943	0.6660	0.0219	0.098*	
H12C	0.6213	0.5825	0.0722	0.098*	
C13	0.3381 (3)	0.5637 (3)	0.2476 (2)	0.0340 (16)*	0.725 (5)
H13	0.3127	0.6004	0.2922	0.041*	0.725 (5)
C14	0.2466 (3)	0.4848 (3)	0.2225 (3)	0.0404 (11)	0.725 (5)
H14A	0.1908	0.5138	0.2041	0.048*	0.725 (5)
H14B	0.2225	0.4484	0.2703	0.048*	0.725 (5)
C15	0.2658 (4)	0.4155 (3)	0.1566 (2)	0.0670 (15)	0.725 (5)
H15A	0.2027	0.3666	0.1443	0.101*	0.725 (5)
H15B	0.3198	0.3850	0.1746	0.101*	0.725 (5)
H15C	0.2874	0.4502	0.1083	0.101*	0.725 (5)
C16	0.4247 (3)	0.5297 (4)	0.2862 (3)	0.0372 (12)	0.725 (5)
H16A	0.4810	0.5847	0.3013	0.056*	0.725 (5)
H16B	0.4489	0.4864	0.2480	0.056*	0.725 (5)
H16C	0.4004	0.4957	0.3344	0.056*	0.725 (5)
C13'	0.3510 (9)	0.5757 (8)	0.2572 (5)	0.027 (3)*	0.275 (5)
H13'	0.3149	0.6094	0.2972	0.033*	0.275 (5)
C14'	0.4455 (9)	0.5609 (11)	0.2977 (8)	0.064 (6)	0.275 (5)
H14C	0.4817	0.5282	0.2580	0.076*	0.275 (5)
H14D	0.4907	0.6246	0.3116	0.076*	0.275 (5)
C15'	0.4339 (7)	0.5057 (7)	0.3715 (5)	0.050 (3)	0.275 (5)
H15D	0.3776	0.4489	0.3631	0.075*	0.275 (5)
H15E	0.4189	0.5458	0.4169	0.075*	0.275 (5)
H15F	0.4976	0.4861	0.3833	0.075*	0.275 (5)
C16'	0.2764 (14)	0.4862 (13)	0.2259 (16)	0.158 (12)	0.275 (5)
H16D	0.3066	0.4564	0.1813	0.237*	0.275 (5)
H16E	0.2132	0.5022	0.2069	0.237*	0.275 (5)
H16F	0.2608	0.4416	0.2691	0.237*	0.275 (5)
C17	0.33347 (18)	1.03520 (17)	0.04694 (14)	0.0329 (5)	
H17	0.3930	1.0462	0.0810	0.040*	
C18	0.33270 (19)	1.09401 (18)	−0.01748 (15)	0.0397 (6)	
H18	0.3911	1.1434	−0.0269	0.048*	
C19	0.24760 (19)	1.07993 (18)	−0.06662 (14)	0.0362 (6)	
H19	0.2453	1.1210	−0.1093	0.043*	
C20	0.16330 (17)	1.00413 (16)	−0.05364 (13)	0.0278 (5)	
C21	0.17103 (16)	0.94775 (15)	0.01207 (12)	0.0228 (5)	
C22	0.07059 (18)	0.98366 (18)	−0.10259 (13)	0.0333 (6)	
H22	0.0654	1.0209	−0.1474	0.040*	
C23	−0.00922 (18)	0.91165 (17)	−0.08521 (13)	0.0315 (5)	
H23	−0.0701	0.8998	−0.1178	0.038*	
C24	−0.00404 (16)	0.85280 (16)	−0.01878 (13)	0.0262 (5)	
C25	0.08632 (15)	0.87023 (15)	0.02919 (12)	0.0216 (4)	
C26	−0.08585 (17)	0.77978 (17)	0.00372 (14)	0.0323 (6)	
H26	−0.1488	0.7656	−0.0264	0.039*	
C27	−0.07429 (17)	0.72921 (17)	0.06938 (14)	0.0322 (5)	
H27	−0.1295	0.6803	0.0857	0.039*	
C28	0.01936 (16)	0.74993 (16)	0.11228 (13)	0.0268 (5)	
H28A	0.0267	0.7136	0.1573	0.032*	
C29	0.70977 (16)	0.80548 (15)	0.33003 (13)	0.0260 (5)	
C30	0.59164 (16)	0.89209 (18)	0.39022 (15)	0.0386 (6)	
H30A	0.5907	0.9605	0.3976	0.046*	
H30B	0.6122	0.8712	0.4425	0.046*	
C31	0.48387 (16)	0.83533 (19)	0.36736 (15)	0.0405 (6)	
H31A	0.4627	0.8557	0.3150	0.049*	
H31B	0.4838	0.7666	0.3609	0.049*	
C32	0.4074 (2)	0.8491 (3)	0.42999 (17)	0.0604 (9)	
H32A	0.3391	0.8113	0.4132	0.091*	
H32B	0.4061	0.9169	0.4356	0.091*	
H32C	0.4275	0.8281	0.4817	0.091*	
C33	0.7071 (4)	0.9658 (3)	0.2849 (3)	0.0330 (17)*	0.587 (6)
H33	0.7536	0.9481	0.2436	0.040*	0.587 (6)
C34	0.7694 (5)	1.0455 (5)	0.3393 (3)	0.048 (2)	0.587 (6)
H34A	0.8180	1.0205	0.3727	0.057*	0.587 (6)
H34B	0.7234	1.0712	0.3756	0.057*	0.587 (6)
C35	0.8275 (6)	1.1239 (5)	0.2903 (4)	0.110 (3)	0.587 (6)
H35A	0.8695	1.1744	0.3262	0.165*	0.587 (6)
H35B	0.7791	1.1505	0.2591	0.165*	0.587 (6)
H35C	0.8719	1.0981	0.2535	0.165*	0.587 (6)
C36	0.6203 (5)	0.9954 (6)	0.2410 (5)	0.0475 (17)	0.587 (6)
H36A	0.5812	0.9411	0.2073	0.071*	0.587 (6)
H36B	0.6486	1.0485	0.2072	0.071*	0.587 (6)
H36C	0.5750	1.0157	0.2803	0.071*	0.587 (6)
C33'	0.6986 (5)	0.9574 (4)	0.2709 (4)	0.032 (2)*	0.413 (6)
H33'	0.7432	0.9326	0.2317	0.039*	0.413 (6)
C34'	0.7623 (6)	1.0510 (6)	0.3072 (6)	0.047 (3)	0.413 (6)
H34C	0.7738	1.0984	0.2651	0.056*	0.413 (6)
H34D	0.7228	1.0749	0.3500	0.056*	0.413 (6)
C35'	0.8640 (5)	1.0453 (6)	0.3423 (5)	0.071 (3)	0.413 (6)
H35D	0.8970	1.1068	0.3698	0.107*	0.413 (6)
H35E	0.9076	1.0305	0.2992	0.107*	0.413 (6)
H35F	0.8541	0.9950	0.3810	0.107*	0.413 (6)
C36'	0.6090 (7)	0.9754 (7)	0.2220 (6)	0.032 (2)*	0.413 (6)
H36D	0.5718	0.9155	0.1944	0.048*	0.413 (6)
H36E	0.6345	1.0225	0.1821	0.048*	0.413 (6)
H36F	0.5627	0.9999	0.2579	0.048*	0.413 (6)
C37	0.79047 (17)	0.49164 (15)	0.20281 (13)	0.0273 (5)	
C38	0.6826 (2)	0.3684 (2)	0.11202 (16)	0.0580 (9)	
H38A	0.6862	0.3480	0.0547	0.070*	
H38B	0.6309	0.4074	0.1159	0.070*	
C39	0.6487 (2)	0.2819 (2)	0.15941 (19)	0.0749 (11)	
H39A	0.6487	0.3012	0.2173	0.090*	
H39B	0.6968	0.2396	0.1523	0.090*	
C40	0.5406 (3)	0.2280 (3)	0.1309 (2)	0.1136 (18)	
H40A	0.5184	0.1715	0.1624	0.170*	
H40B	0.5411	0.2082	0.0739	0.170*	
H40C	0.4931	0.2701	0.1384	0.170*	
C41	0.8671 (4)	0.4267 (4)	0.0836 (3)	0.0364 (15)	0.557 (6)
H41	0.9292	0.4751	0.1045	0.044*	0.557 (6)
C42	0.8886 (4)	0.3293 (4)	0.0892 (3)	0.0545 (18)	0.557 (6)
H42A	0.9471	0.3258	0.0547	0.065*	0.557 (6)
H42B	0.8282	0.2812	0.0668	0.065*	0.557 (6)
C43	0.9128 (9)	0.3019 (7)	0.1721 (5)	0.066 (3)	0.557 (6)
H43A	0.9247	0.2371	0.1691	0.100*	0.557 (6)
H43B	0.8551	0.3036	0.2070	0.100*	0.557 (6)
H43C	0.9744	0.3470	0.1943	0.100*	0.557 (6)
C44	0.8444 (9)	0.4498 (6)	−0.0015 (4)	0.046 (2)	0.557 (6)
H44A	0.8313	0.5140	−0.0013	0.069*	0.557 (6)
H44B	0.7838	0.4030	−0.0234	0.069*	0.557 (6)
H44C	0.9032	0.4473	−0.0349	0.069*	0.557 (6)
C41'	0.8808 (5)	0.4012 (6)	0.1153 (3)	0.045 (2)	0.443 (6)
H41'	0.9334	0.4630	0.1218	0.054*	0.443 (6)
C42'	0.8747 (5)	0.3790 (5)	0.0256 (3)	0.053 (2)	0.443 (6)
H42C	0.9399	0.3643	0.0086	0.064*	0.443 (6)
H42D	0.8193	0.3211	0.0132	0.064*	0.443 (6)
C43'	0.8546 (13)	0.4592 (9)	−0.0221 (6)	0.062 (4)	0.443 (6)
H43D	0.8591	0.5161	0.0138	0.093*	0.443 (6)
H43E	0.7860	0.4401	−0.0474	0.093*	0.443 (6)
H43F	0.9058	0.4739	−0.0638	0.093*	0.443 (6)
C44'	0.9294 (11)	0.3322 (8)	0.1591 (6)	0.062 (3)	0.443 (6)
H44D	0.8851	0.2676	0.1525	0.093*	0.443 (6)
H44E	0.9380	0.3519	0.2165	0.093*	0.443 (6)
H44F	0.9964	0.3323	0.1367	0.093*	0.443 (6)
C45	0.73245 (19)	0.47705 (18)	0.45705 (15)	0.0377 (6)	
H45	0.6738	0.4561	0.4224	0.045*	
C46	0.7400 (2)	0.4268 (2)	0.52591 (16)	0.0456 (7)	
H46	0.6870	0.3732	0.5374	0.055*	
C47	0.8238 (2)	0.45501 (19)	0.57653 (15)	0.0408 (7)	
H47	0.8302	0.4210	0.6231	0.049*	
C48	0.90100 (19)	0.53560 (18)	0.55858 (13)	0.0329 (6)	
C49	0.88660 (17)	0.58166 (16)	0.48816 (12)	0.0253 (5)	
C50	0.9926 (2)	0.5698 (2)	0.60707 (14)	0.0389 (6)	
H50	1.0019	0.5391	0.6550	0.047*	
C51	1.0654 (2)	0.6444 (2)	0.58583 (14)	0.0389 (6)	
H51	1.1258	0.6651	0.6187	0.047*	
C52	1.05418 (17)	0.69385 (17)	0.51424 (13)	0.0295 (5)	
C53	0.96394 (16)	0.66307 (16)	0.46640 (12)	0.0230 (5)	
C54	1.12855 (18)	0.77028 (19)	0.48772 (14)	0.0360 (6)	
H54	1.1909	0.7931	0.5178	0.043*	
C55	1.11122 (17)	0.81217 (17)	0.41822 (14)	0.0331 (5)	
H55	1.1619	0.8633	0.3993	0.040*	
C56	1.01803 (16)	0.77887 (16)	0.37545 (13)	0.0276 (5)	
H56	1.0059	0.8095	0.3281	0.033*	

### Atomic displacement parameters (Å^2^)

**Table:**

	*U*^11^	*U*^22^	*U*^33^	*U*^12^	*U*^13^	*U*^23^
Zn1	0.02242 (14)	0.01901 (13)	0.02348 (13)	0.00553 (10)	−0.00139 (10)	0.00109 (10)
Zn2	0.02268 (14)	0.02026 (14)	0.02312 (14)	0.00700 (10)	−0.00043 (10)	0.00007 (10)
S1	0.0223 (3)	0.0292 (3)	0.0326 (3)	0.0100 (2)	−0.0038 (2)	−0.0026 (2)
S2	0.0220 (3)	0.0253 (3)	0.0270 (3)	0.0048 (2)	−0.0031 (2)	−0.0052 (2)
S3	0.0270 (3)	0.0299 (3)	0.0344 (3)	0.0102 (2)	0.0060 (2)	0.0094 (3)
S4	0.0375 (3)	0.0279 (3)	0.0285 (3)	0.0150 (3)	0.0082 (3)	0.0073 (2)
S5	0.0269 (3)	0.0256 (3)	0.0352 (3)	0.0124 (2)	0.0033 (2)	0.0047 (2)
S6	0.0309 (3)	0.0336 (3)	0.0273 (3)	0.0149 (3)	0.0017 (2)	0.0003 (2)
S7	0.0224 (3)	0.0293 (3)	0.0374 (3)	0.0065 (2)	0.0035 (2)	−0.0054 (3)
S8	0.0227 (3)	0.0235 (3)	0.0271 (3)	0.0041 (2)	0.0004 (2)	−0.0031 (2)
N1	0.0226 (10)	0.0339 (12)	0.0341 (11)	0.0005 (9)	0.0029 (8)	−0.0131 (9)
N2	0.0361 (11)	0.0242 (10)	0.0304 (10)	0.0119 (9)	0.0076 (9)	0.0037 (8)
N3	0.0241 (10)	0.0224 (10)	0.0264 (10)	0.0056 (8)	−0.0008 (8)	0.0026 (8)
N4	0.0230 (9)	0.0203 (9)	0.0219 (9)	0.0055 (8)	0.0009 (7)	−0.0021 (7)
N5	0.0258 (10)	0.0263 (11)	0.0368 (11)	0.0123 (8)	−0.0015 (8)	−0.0046 (8)
N6	0.0287 (11)	0.0439 (14)	0.0523 (14)	−0.0080 (10)	0.0143 (10)	−0.0273 (11)
N7	0.0278 (10)	0.0255 (10)	0.0266 (10)	0.0099 (8)	0.0054 (8)	0.0024 (8)
N8	0.0234 (9)	0.0229 (10)	0.0227 (9)	0.0097 (8)	0.0018 (7)	−0.0017 (7)
C1	0.0253 (11)	0.0206 (11)	0.0203 (10)	0.0031 (9)	0.0008 (9)	0.0032 (9)
C2	0.0275 (13)	0.0417 (15)	0.0266 (12)	−0.0032 (11)	−0.0007 (10)	−0.0124 (10)
C3	0.0458 (16)	0.0363 (15)	0.0463 (16)	−0.0059 (12)	−0.0011 (13)	−0.0011 (12)
C4	0.0567 (19)	0.071 (2)	0.066 (2)	−0.0306 (17)	−0.0016 (16)	0.0080 (18)
C5	0.026 (3)	0.046 (4)	0.053 (4)	−0.006 (3)	0.019 (3)	−0.024 (3)
C6	0.047 (3)	0.063 (4)	0.069 (4)	0.029 (3)	−0.009 (3)	−0.031 (3)
C7	0.094 (5)	0.061 (5)	0.086 (6)	0.044 (4)	−0.031 (5)	0.001 (4)
C8	0.051 (4)	0.061 (6)	0.051 (5)	−0.001 (5)	0.024 (4)	−0.011 (4)
C5'	0.027 (3)	0.030 (4)	0.043 (4)	0.004 (3)	0.009 (3)	−0.013 (3)
C6'	0.035 (3)	0.038 (4)	0.040 (4)	0.014 (3)	0.002 (3)	−0.007 (3)
C7'	0.049 (5)	0.052 (5)	0.042 (5)	0.014 (4)	0.010 (4)	0.012 (4)
C8'	0.077 (6)	0.070 (5)	0.060 (6)	0.055 (4)	0.007 (6)	0.020 (5)
C9	0.0271 (12)	0.0204 (11)	0.0262 (11)	0.0066 (9)	−0.0014 (9)	0.0003 (9)
C10	0.0447 (15)	0.0330 (14)	0.0461 (15)	0.0199 (12)	0.0108 (12)	0.0066 (12)
C11	0.0398 (15)	0.0392 (16)	0.0599 (18)	0.0149 (12)	0.0053 (13)	0.0117 (13)
C12	0.0481 (18)	0.060 (2)	0.098 (3)	0.0249 (16)	0.0317 (18)	0.0305 (19)
C14	0.042 (2)	0.025 (2)	0.053 (3)	0.0022 (16)	0.0203 (19)	0.0114 (17)
C15	0.079 (3)	0.045 (3)	0.069 (3)	0.002 (2)	−0.005 (2)	−0.003 (2)
C16	0.034 (2)	0.031 (3)	0.050 (3)	0.010 (2)	0.0083 (19)	0.0180 (19)
C14'	0.044 (7)	0.057 (9)	0.078 (8)	−0.021 (6)	−0.008 (6)	0.040 (7)
C15'	0.049 (6)	0.050 (6)	0.059 (6)	0.024 (5)	0.015 (5)	0.017 (5)
C16'	0.170 (15)	0.151 (15)	0.141 (14)	0.014 (10)	−0.012 (9)	0.014 (9)
C17	0.0285 (13)	0.0287 (13)	0.0394 (14)	0.0013 (10)	−0.0022 (11)	0.0066 (11)
C18	0.0398 (15)	0.0341 (15)	0.0423 (15)	−0.0001 (12)	0.0058 (12)	0.0140 (12)
C19	0.0452 (15)	0.0341 (14)	0.0313 (13)	0.0108 (12)	0.0070 (11)	0.0131 (11)
C20	0.0354 (13)	0.0298 (13)	0.0221 (11)	0.0158 (11)	0.0013 (10)	0.0006 (9)
C21	0.0260 (12)	0.0241 (12)	0.0208 (11)	0.0110 (9)	0.0024 (9)	−0.0004 (9)
C22	0.0429 (15)	0.0420 (15)	0.0216 (11)	0.0234 (12)	−0.0022 (10)	0.0012 (10)
C23	0.0330 (13)	0.0389 (14)	0.0263 (12)	0.0189 (11)	−0.0076 (10)	−0.0083 (10)
C24	0.0268 (12)	0.0290 (13)	0.0251 (11)	0.0134 (10)	−0.0037 (9)	−0.0085 (9)
C25	0.0229 (11)	0.0217 (11)	0.0217 (11)	0.0092 (9)	0.0007 (9)	−0.0043 (9)
C26	0.0225 (12)	0.0352 (14)	0.0390 (14)	0.0099 (10)	−0.0056 (10)	−0.0137 (11)
C27	0.0234 (12)	0.0302 (13)	0.0401 (14)	0.0021 (10)	0.0029 (10)	−0.0070 (11)
C28	0.0265 (12)	0.0233 (12)	0.0294 (12)	0.0039 (10)	0.0030 (10)	−0.0027 (9)
C29	0.0219 (11)	0.0245 (12)	0.0310 (12)	0.0065 (9)	−0.0065 (9)	−0.0057 (9)
C30	0.0385 (14)	0.0426 (15)	0.0417 (14)	0.0246 (12)	0.0040 (12)	−0.0043 (12)
C31	0.0315 (14)	0.0462 (16)	0.0489 (16)	0.0175 (12)	0.0086 (12)	0.0094 (13)
C32	0.0468 (17)	0.091 (3)	0.0545 (18)	0.0339 (17)	0.0156 (14)	0.0254 (17)
C34	0.051 (4)	0.037 (3)	0.048 (4)	−0.005 (3)	−0.006 (3)	0.001 (3)
C35	0.143 (7)	0.078 (5)	0.105 (5)	0.017 (5)	0.009 (5)	0.007 (4)
C36	0.050 (3)	0.042 (4)	0.057 (4)	0.022 (3)	0.001 (3)	0.000 (3)
C34'	0.044 (5)	0.040 (5)	0.052 (6)	0.001 (4)	0.010 (4)	−0.008 (4)
C35'	0.084 (6)	0.059 (5)	0.065 (5)	0.008 (4)	0.001 (4)	−0.012 (4)
C37	0.0262 (12)	0.0230 (12)	0.0309 (12)	0.0030 (10)	0.0040 (10)	−0.0029 (10)
C38	0.0557 (18)	0.058 (2)	0.0447 (17)	−0.0132 (15)	0.0106 (14)	−0.0243 (14)
C39	0.083 (2)	0.062 (2)	0.064 (2)	−0.0119 (19)	0.0188 (19)	−0.0179 (18)
C40	0.090 (3)	0.132 (4)	0.068 (2)	−0.074 (3)	0.020 (2)	−0.030 (2)
C41	0.038 (3)	0.039 (3)	0.027 (3)	0.000 (2)	0.015 (2)	−0.006 (2)
C42	0.058 (3)	0.062 (4)	0.049 (4)	0.028 (3)	0.008 (3)	−0.017 (3)
C43	0.087 (6)	0.060 (6)	0.069 (5)	0.053 (5)	−0.008 (4)	−0.006 (4)
C44	0.048 (4)	0.048 (4)	0.030 (3)	−0.013 (3)	0.022 (4)	−0.004 (3)
C41'	0.038 (4)	0.050 (5)	0.044 (4)	0.011 (3)	0.010 (3)	−0.023 (4)
C42'	0.042 (4)	0.062 (5)	0.052 (4)	0.012 (3)	−0.004 (3)	−0.020 (4)
C43'	0.054 (6)	0.081 (7)	0.051 (6)	0.012 (5)	0.032 (5)	−0.004 (5)
C44'	0.068 (5)	0.049 (6)	0.069 (6)	0.015 (5)	0.011 (5)	−0.011 (4)
C45	0.0364 (14)	0.0336 (14)	0.0424 (15)	0.0045 (11)	0.0082 (12)	0.0099 (12)
C46	0.0530 (18)	0.0359 (15)	0.0495 (17)	0.0093 (13)	0.0200 (14)	0.0184 (13)
C47	0.0585 (18)	0.0406 (16)	0.0337 (14)	0.0283 (14)	0.0205 (13)	0.0171 (12)
C48	0.0450 (15)	0.0402 (15)	0.0238 (12)	0.0291 (12)	0.0102 (11)	0.0060 (10)
C49	0.0302 (12)	0.0283 (12)	0.0226 (11)	0.0171 (10)	0.0064 (9)	0.0014 (9)
C50	0.0533 (17)	0.0552 (18)	0.0201 (12)	0.0364 (14)	0.0053 (11)	0.0042 (11)
C51	0.0392 (15)	0.0610 (19)	0.0250 (12)	0.0316 (14)	−0.0050 (11)	−0.0066 (12)
C52	0.0298 (13)	0.0397 (14)	0.0229 (11)	0.0180 (11)	−0.0003 (10)	−0.0079 (10)
C53	0.0250 (11)	0.0291 (12)	0.0193 (10)	0.0160 (10)	0.0022 (9)	−0.0039 (9)
C54	0.0242 (12)	0.0494 (16)	0.0344 (13)	0.0125 (11)	−0.0071 (10)	−0.0152 (12)
C55	0.0236 (12)	0.0325 (14)	0.0412 (14)	0.0037 (10)	0.0016 (11)	−0.0059 (11)
C56	0.0266 (12)	0.0270 (12)	0.0287 (12)	0.0062 (10)	0.0011 (10)	−0.0016 (10)

### Geometric parameters (Å, °)

**Table:**

Zn1—N3	2.1998 (17)	C16'—H16E	0.9800
Zn1—N4	2.2133 (17)	C16'—H16F	0.9800
Zn1—S3	2.4912 (6)	C17—C18	1.399 (3)
Zn1—S4	2.4930 (6)	C17—H17	0.9500
Zn1—S1	2.5073 (6)	C18—C19	1.363 (3)
Zn1—S2	2.5311 (6)	C18—H18	0.9500
Zn2—N8	2.2106 (17)	C19—C20	1.406 (3)
Zn2—N7	2.2272 (18)	C19—H19	0.9500
Zn2—S6	2.4881 (6)	C20—C21	1.409 (3)
Zn2—S7	2.4964 (6)	C20—C22	1.439 (3)
Zn2—S5	2.5129 (6)	C21—C25	1.440 (3)
Zn2—S8	2.5178 (6)	C22—C23	1.353 (3)
S1—C1	1.717 (2)	C22—H22	0.9500
S2—C1	1.719 (2)	C23—C24	1.435 (3)
S3—C9	1.725 (2)	C23—H23	0.9500
S4—C9	1.718 (2)	C24—C26	1.403 (3)
S5—C29	1.727 (2)	C24—C25	1.406 (3)
S6—C29	1.721 (2)	C26—C27	1.368 (3)
S7—C37	1.715 (2)	C26—H26	0.9500
S8—C37	1.720 (2)	C27—C28	1.398 (3)
N1—C1	1.341 (2)	C27—H27	0.9500
N1—C2	1.471 (2)	C28—H28A	0.9500
N1—C5	1.483 (4)	C30—C31	1.520 (3)
N1—C5'	1.491 (4)	C30—H30A	0.9900
N2—C9	1.342 (2)	C30—H30B	0.9900
N2—C10	1.454 (3)	C31—C32	1.513 (3)
N2—C13	1.479 (3)	C31—H31A	0.9900
N2—C13'	1.478 (5)	C31—H31B	0.9900
N3—C17	1.329 (3)	C32—H32A	0.9800
N3—C21	1.352 (3)	C32—H32B	0.9800
N4—C28	1.324 (3)	C32—H32C	0.9800
N4—C25	1.353 (3)	C33—C34	1.504 (4)
N5—C29	1.344 (2)	C33—C36	1.517 (4)
N5—C33	1.455 (4)	C33—H33	1.0000
N5—C30	1.465 (2)	C34—C35	1.501 (5)
N5—C33'	1.502 (4)	C34—H34A	0.9900
N6—C37	1.346 (2)	C34—H34B	0.9900
N6—C41	1.476 (4)	C35—H35A	0.9800
N6—C38	1.479 (3)	C35—H35B	0.9800
N6—C41'	1.502 (4)	C35—H35C	0.9800
N7—C45	1.324 (3)	C36—H36A	0.9800
N7—C49	1.354 (3)	C36—H36B	0.9800
N8—C56	1.329 (3)	C36—H36C	0.9800
N8—C53	1.356 (3)	C33'—C34'	1.511 (5)
C2—C3	1.510 (3)	C33'—C36'	1.519 (5)
C2—H2A	0.9900	C33'—H33'	1.0000
C2—H2B	0.9900	C34'—C35'	1.496 (5)
C3—C4	1.508 (3)	C34'—H34C	0.9900
C3—H3A	0.9900	C34'—H34D	0.9900
C3—H3B	0.9900	C35'—H35D	0.9800
C4—H4A	0.9800	C35'—H35E	0.9800
C4—H4B	0.9800	C35'—H35F	0.9800
C4—H4C	0.9800	C36'—H36D	0.9800
C5—C8	1.504 (5)	C36'—H36E	0.9800
C5—C6	1.510 (4)	C36'—H36F	0.9800
C5—H5	1.0000	C38—C39	1.491 (3)
C6—C7	1.513 (5)	C38—H38A	0.9900
C6—H6A	0.9900	C38—H38B	0.9900
C6—H6B	0.9900	C39—C40	1.534 (3)
C7—H7A	0.9800	C39—H39A	0.9900
C7—H7B	0.9800	C39—H39B	0.9900
C7—H7C	0.9800	C40—H40A	0.9800
C8—H8A	0.9800	C40—H40B	0.9800
C8—H8B	0.9800	C40—H40C	0.9800
C8—H8C	0.9800	C41—C42	1.502 (4)
C5'—C6'	1.511 (5)	C41—C44	1.511 (5)
C5'—C8'	1.514 (5)	C41—H41	1.0000
C5'—H5'	1.0000	C42—C43	1.504 (5)
C6'—C7'	1.503 (5)	C42—H42A	0.9900
C6'—H6'A	0.9900	C42—H42B	0.9900
C6'—H6'B	0.9900	C43—H43A	0.9800
C7'—H7'A	0.9800	C43—H43B	0.9800
C7'—H7'B	0.9800	C43—H43C	0.9800
C7'—H7'C	0.9800	C44—H44A	0.9800
C8'—H8'A	0.9800	C44—H44B	0.9800
C8'—H8'B	0.9800	C44—H44C	0.9800
C8'—H8'C	0.9800	C41'—C42'	1.509 (4)
C10—C11	1.481 (3)	C41'—C44'	1.515 (5)
C10—H10A	0.9900	C41'—H41'	1.0000
C10—H10B	0.9900	C42'—C43'	1.504 (5)
C11—C12	1.552 (3)	C42'—H42C	0.9900
C11—H11A	0.9900	C42'—H42D	0.9900
C11—H11B	0.9900	C43'—H43D	0.9800
C12—H12A	0.9800	C43'—H43E	0.9800
C12—H12B	0.9800	C43'—H43F	0.9800
C12—H12C	0.9800	C44'—H44D	0.9800
C13—C14	1.505 (4)	C44'—H44E	0.9800
C13—C16	1.508 (4)	C44'—H44F	0.9800
C13—H13	1.0000	C45—C46	1.399 (3)
C14—C15	1.512 (4)	C45—H45	0.9500
C14—H14A	0.9900	C46—C47	1.365 (4)
C14—H14B	0.9900	C46—H46	0.9500
C15—H15A	0.9800	C47—C48	1.413 (4)
C15—H15B	0.9800	C47—H47	0.9500
C15—H15C	0.9800	C48—C49	1.406 (3)
C16—H16A	0.9800	C48—C50	1.433 (4)
C16—H16B	0.9800	C49—C53	1.440 (3)
C16—H16C	0.9800	C50—C51	1.340 (4)
C13'—C14'	1.489 (5)	C50—H50	0.9500
C13'—C16'	1.502 (5)	C51—C52	1.440 (3)
C13'—H13'	1.0000	C51—H51	0.9500
C14'—C15'	1.487 (5)	C52—C54	1.398 (3)
C14'—H14C	0.9900	C52—C53	1.408 (3)
C14'—H14D	0.9900	C54—C55	1.370 (3)
C15'—H15D	0.9800	C54—H54	0.9500
C15'—H15E	0.9800	C55—C56	1.400 (3)
C15'—H15F	0.9800	C55—H55	0.9500
C16'—H16D	0.9800	C56—H56	0.9500
			
N3—Zn1—N4	74.71 (6)	N3—C17—H17	118.7
N3—Zn1—S3	157.35 (5)	C18—C17—H17	118.7
N4—Zn1—S3	96.54 (5)	C19—C18—C17	119.6 (2)
N3—Zn1—S4	89.08 (5)	C19—C18—H18	120.2
N4—Zn1—S4	102.50 (5)	C17—C18—H18	120.2
S3—Zn1—S4	72.192 (19)	C18—C19—C20	119.5 (2)
N3—Zn1—S1	94.18 (5)	C18—C19—H19	120.3
N4—Zn1—S1	151.09 (5)	C20—C19—H19	120.3
S3—Zn1—S1	102.47 (2)	C19—C20—C21	117.2 (2)
S4—Zn1—S1	103.93 (2)	C19—C20—C22	123.3 (2)
N3—Zn1—S2	93.96 (5)	C21—C20—C22	119.5 (2)
N4—Zn1—S2	82.85 (5)	N3—C21—C20	122.9 (2)
S3—Zn1—S2	105.81 (2)	N3—C21—C25	117.40 (18)
S4—Zn1—S2	174.40 (2)	C20—C21—C25	119.6 (2)
S1—Zn1—S2	71.186 (18)	C23—C22—C20	120.4 (2)
N8—Zn2—N7	74.40 (7)	C23—C22—H22	119.8
N8—Zn2—S6	102.68 (5)	C20—C22—H22	119.8
N7—Zn2—S6	89.67 (5)	C22—C23—C24	121.6 (2)
N8—Zn2—S7	153.90 (5)	C22—C23—H23	119.2
N7—Zn2—S7	93.72 (5)	C24—C23—H23	119.2
S6—Zn2—S7	100.36 (2)	C26—C24—C25	117.0 (2)
N8—Zn2—S5	93.35 (5)	C26—C24—C23	123.8 (2)
N7—Zn2—S5	155.36 (5)	C25—C24—C23	119.1 (2)
S6—Zn2—S5	71.94 (2)	N4—C25—C24	123.2 (2)
S7—Zn2—S5	105.38 (2)	N4—C25—C21	117.05 (18)
N8—Zn2—S8	87.39 (5)	C24—C25—C21	119.71 (19)
N7—Zn2—S8	99.97 (5)	C27—C26—C24	119.5 (2)
S6—Zn2—S8	167.69 (2)	C27—C26—H26	120.3
S7—Zn2—S8	71.587 (19)	C24—C26—H26	120.3
S5—Zn2—S8	100.75 (2)	C26—C27—C28	119.5 (2)
C1—S1—Zn1	86.21 (7)	C26—C27—H27	120.2
C1—S2—Zn1	85.43 (7)	C28—C27—H27	120.2
C9—S3—Zn1	85.23 (7)	N4—C28—C27	122.6 (2)
C9—S4—Zn1	85.31 (7)	N4—C28—H28A	118.7
C29—S5—Zn2	85.15 (7)	C27—C28—H28A	118.7
C29—S6—Zn2	86.06 (7)	N5—C29—S6	121.38 (16)
C37—S7—Zn2	85.90 (7)	N5—C29—S5	121.80 (17)
C37—S8—Zn2	85.13 (7)	S6—C29—S5	116.82 (12)
C1—N1—C2	120.16 (18)	N5—C30—C31	114.43 (19)
C1—N1—C5	120.8 (3)	N5—C30—H30A	108.7
C2—N1—C5	117.9 (3)	C31—C30—H30A	108.7
C1—N1—C5'	121.6 (4)	N5—C30—H30B	108.7
C2—N1—C5'	116.8 (4)	C31—C30—H30B	108.7
C9—N2—C10	120.25 (18)	H30A—C30—H30B	107.6
C9—N2—C13	121.4 (2)	C32—C31—C30	112.0 (2)
C10—N2—C13	117.8 (2)	C32—C31—H31A	109.2
C9—N2—C13'	120.2 (5)	C30—C31—H31A	109.2
C10—N2—C13'	119.4 (5)	C32—C31—H31B	109.2
C17—N3—C21	118.16 (19)	C30—C31—H31B	109.2
C17—N3—Zn1	126.42 (15)	H31A—C31—H31B	107.9
C21—N3—Zn1	115.32 (13)	C31—C32—H32A	109.5
C28—N4—C25	118.08 (18)	C31—C32—H32B	109.5
C28—N4—Zn1	125.97 (14)	H32A—C32—H32B	109.5
C25—N4—Zn1	114.93 (13)	C31—C32—H32C	109.5
C29—N5—C33	123.7 (3)	H32A—C32—H32C	109.5
C29—N5—C30	120.03 (19)	H32B—C32—H32C	109.5
C33—N5—C30	115.6 (3)	N5—C33—C34	109.6 (4)
C29—N5—C33'	120.8 (4)	N5—C33—C36	111.2 (5)
C30—N5—C33'	119.2 (4)	C34—C33—C36	112.4 (5)
C37—N6—C41	124.0 (3)	N5—C33—H33	107.8
C37—N6—C38	120.24 (19)	C34—C33—H33	107.8
C41—N6—C38	114.5 (3)	C36—C33—H33	107.8
C37—N6—C41'	116.8 (3)	C33—C34—C35	110.2 (4)
C38—N6—C41'	120.7 (3)	C33—C34—H34A	109.6
C45—N7—C49	117.9 (2)	C35—C34—H34A	109.6
C45—N7—Zn2	126.87 (16)	C33—C34—H34B	109.6
C49—N7—Zn2	115.19 (14)	C35—C34—H34B	109.6
C56—N8—C53	118.30 (19)	H34A—C34—H34B	108.1
C56—N8—Zn2	125.75 (14)	C34—C35—H35A	109.5
C53—N8—Zn2	115.78 (14)	C34—C35—H35B	109.5
N1—C1—S1	121.14 (16)	H35A—C35—H35B	109.5
N1—C1—S2	121.67 (16)	C34—C35—H35C	109.5
S1—C1—S2	117.17 (12)	H35A—C35—H35C	109.5
N1—C2—C3	114.70 (19)	H35B—C35—H35C	109.5
N1—C2—H2A	108.6	C33—C36—H36A	109.5
C3—C2—H2A	108.6	C33—C36—H36B	109.5
N1—C2—H2B	108.6	H36A—C36—H36B	109.5
C3—C2—H2B	108.6	C33—C36—H36C	109.5
H2A—C2—H2B	107.6	H36A—C36—H36C	109.5
C4—C3—C2	111.4 (2)	H36B—C36—H36C	109.5
C4—C3—H3A	109.4	N5—C33'—C34'	113.2 (6)
C2—C3—H3A	109.4	N5—C33'—C36'	114.1 (6)
C4—C3—H3B	109.4	C34'—C33'—C36'	109.8 (5)
C2—C3—H3B	109.4	N5—C33'—H33'	106.4
H3A—C3—H3B	108.0	C34'—C33'—H33'	106.4
C3—C4—H4A	109.5	C36'—C33'—H33'	106.4
C3—C4—H4B	109.5	C35'—C34'—C33'	114.6 (6)
H4A—C4—H4B	109.5	C35'—C34'—H34C	108.6
C3—C4—H4C	109.5	C33'—C34'—H34C	108.6
H4A—C4—H4C	109.5	C35'—C34'—H34D	108.6
H4B—C4—H4C	109.5	C33'—C34'—H34D	108.6
N1—C5—C8	114.4 (6)	H34C—C34'—H34D	107.6
N1—C5—C6	106.3 (4)	C34'—C35'—H35D	109.5
C8—C5—C6	113.1 (5)	C34'—C35'—H35E	109.5
N1—C5—H5	107.6	H35D—C35'—H35E	109.5
C8—C5—H5	107.6	C34'—C35'—H35F	109.5
C6—C5—H5	107.6	H35D—C35'—H35F	109.5
C5—C6—C7	114.4 (5)	H35E—C35'—H35F	109.5
C5—C6—H6A	108.7	C33'—C36'—H36D	109.5
C7—C6—H6A	108.7	C33'—C36'—H36E	109.5
C5—C6—H6B	108.7	H36D—C36'—H36E	109.5
C7—C6—H6B	108.7	C33'—C36'—H36F	109.5
H6A—C6—H6B	107.6	H36D—C36'—H36F	109.5
C6—C7—H7A	109.5	H36E—C36'—H36F	109.5
C6—C7—H7B	109.5	N6—C37—S7	121.07 (16)
H7A—C7—H7B	109.5	N6—C37—S8	121.64 (16)
C6—C7—H7C	109.5	S7—C37—S8	117.26 (12)
H7A—C7—H7C	109.5	N6—C38—C39	113.0 (2)
H7B—C7—H7C	109.5	N6—C38—H38A	109.0
C5—C8—H8A	109.5	C39—C38—H38A	109.0
C5—C8—H8B	109.5	N6—C38—H38B	109.0
H8A—C8—H8B	109.5	C39—C38—H38B	109.0
C5—C8—H8C	109.5	H38A—C38—H38B	107.8
H8A—C8—H8C	109.5	C38—C39—C40	109.7 (3)
H8B—C8—H8C	109.5	C38—C39—H39A	109.7
N1—C5'—C6'	111.4 (4)	C40—C39—H39A	109.7
N1—C5'—C8'	120.0 (6)	C38—C39—H39B	109.7
C6'—C5'—C8'	112.2 (5)	C40—C39—H39B	109.7
N1—C5'—H5'	103.7	H39A—C39—H39B	108.2
C6'—C5'—H5'	103.7	C39—C40—H40A	109.5
C8'—C5'—H5'	103.7	C39—C40—H40B	109.5
C7'—C6'—C5'	113.6 (5)	H40A—C40—H40B	109.5
C7'—C6'—H6'A	108.8	C39—C40—H40C	109.5
C5'—C6'—H6'A	108.8	H40A—C40—H40C	109.5
C7'—C6'—H6'B	108.8	H40B—C40—H40C	109.5
C5'—C6'—H6'B	108.8	N6—C41—C42	105.0 (3)
H6'A—C6'—H6'B	107.7	N6—C41—C44	114.4 (6)
C6'—C7'—H7'A	109.5	C42—C41—C44	113.3 (5)
C6'—C7'—H7'B	109.5	N6—C41—H41	108.0
H7'A—C7'—H7'B	109.5	C42—C41—H41	108.0
C6'—C7'—H7'C	109.5	C44—C41—H41	108.0
H7'A—C7'—H7'C	109.5	C41—C42—C43	116.1 (5)
H7'B—C7'—H7'C	109.5	C41—C42—H42A	108.3
C5'—C8'—H8'A	109.5	C43—C42—H42A	108.3
C5'—C8'—H8'B	109.5	C41—C42—H42B	108.3
H8'A—C8'—H8'B	109.5	C43—C42—H42B	108.3
C5'—C8'—H8'C	109.5	H42A—C42—H42B	107.4
H8'A—C8'—H8'C	109.5	C42—C43—H43A	109.5
H8'B—C8'—H8'C	109.5	C42—C43—H43B	109.5
N2—C9—S4	121.47 (16)	H43A—C43—H43B	109.5
N2—C9—S3	121.44 (16)	C42—C43—H43C	109.5
S4—C9—S3	117.08 (12)	H43A—C43—H43C	109.5
N2—C10—C11	114.7 (2)	H43B—C43—H43C	109.5
N2—C10—H10A	108.6	C41—C44—H44A	109.5
C11—C10—H10A	108.6	C41—C44—H44B	109.5
N2—C10—H10B	108.6	H44A—C44—H44B	109.5
C11—C10—H10B	108.6	C41—C44—H44C	109.5
H10A—C10—H10B	107.6	H44A—C44—H44C	109.5
C10—C11—C12	110.7 (2)	H44B—C44—H44C	109.5
C10—C11—H11A	109.5	N6—C41'—C42'	108.3 (4)
C12—C11—H11A	109.5	N6—C41'—C44'	124.3 (7)
C10—C11—H11B	109.5	C42'—C41'—C44'	111.4 (5)
C12—C11—H11B	109.5	N6—C41'—H41'	103.5
H11A—C11—H11B	108.1	C42'—C41'—H41'	103.5
C11—C12—H12A	109.5	C44'—C41'—H41'	103.5
C11—C12—H12B	109.5	C43'—C42'—C41'	113.4 (6)
H12A—C12—H12B	109.5	C43'—C42'—H42C	108.9
C11—C12—H12C	109.5	C41'—C42'—H42C	108.9
H12A—C12—H12C	109.5	C43'—C42'—H42D	108.9
H12B—C12—H12C	109.5	C41'—C42'—H42D	108.9
N2—C13—C14	115.2 (3)	H42C—C42'—H42D	107.7
N2—C13—C16	113.5 (3)	C42'—C43'—H43D	109.5
C14—C13—C16	114.0 (3)	C42'—C43'—H43E	109.5
N2—C13—H13	104.1	H43D—C43'—H43E	109.5
C14—C13—H13	104.1	C42'—C43'—H43F	109.5
C16—C13—H13	104.1	H43D—C43'—H43F	109.5
C13—C14—C15	114.9 (4)	H43E—C43'—H43F	109.5
C13—C14—H14A	108.5	C41'—C44'—H44D	109.5
C15—C14—H14A	108.5	C41'—C44'—H44E	109.5
C13—C14—H14B	108.5	H44D—C44'—H44E	109.5
C15—C14—H14B	108.5	C41'—C44'—H44F	109.5
H14A—C14—H14B	107.5	H44D—C44'—H44F	109.5
C14—C15—H15A	109.5	H44E—C44'—H44F	109.5
C14—C15—H15B	109.5	N7—C45—C46	122.9 (3)
H15A—C15—H15B	109.5	N7—C45—H45	118.6
C14—C15—H15C	109.5	C46—C45—H45	118.6
H15A—C15—H15C	109.5	C47—C46—C45	119.7 (2)
H15B—C15—H15C	109.5	C47—C46—H46	120.1
C13—C16—H16A	109.5	C45—C46—H46	120.1
C13—C16—H16B	109.5	C46—C47—C48	119.0 (2)
H16A—C16—H16B	109.5	C46—C47—H47	120.5
C13—C16—H16C	109.5	C48—C47—H47	120.5
H16A—C16—H16C	109.5	C49—C48—C47	117.2 (2)
H16B—C16—H16C	109.5	C49—C48—C50	119.6 (2)
N2—C13'—C14'	113.2 (7)	C47—C48—C50	123.2 (2)
N2—C13'—C16'	104.6 (12)	N7—C49—C48	123.3 (2)
C14'—C13'—C16'	115.4 (8)	N7—C49—C53	117.43 (18)
N2—C13'—H13'	107.8	C48—C49—C53	119.3 (2)
C14'—C13'—H13'	107.8	C51—C50—C48	121.0 (2)
C16'—C13'—H13'	107.8	C51—C50—H50	119.5
C15'—C14'—C13'	118.0 (7)	C48—C50—H50	119.5
C15'—C14'—H14C	107.8	C50—C51—C52	121.5 (2)
C13'—C14'—H14C	107.8	C50—C51—H51	119.3
C15'—C14'—H14D	107.8	C52—C51—H51	119.3
C13'—C14'—H14D	107.8	C54—C52—C53	117.1 (2)
H14C—C14'—H14D	107.2	C54—C52—C51	124.1 (2)
C14'—C15'—H15D	109.5	C53—C52—C51	118.7 (2)
C14'—C15'—H15E	109.5	N8—C53—C52	122.9 (2)
H15D—C15'—H15E	109.5	N8—C53—C49	117.18 (19)
C14'—C15'—H15F	109.5	C52—C53—C49	119.9 (2)
H15D—C15'—H15F	109.5	C55—C54—C52	119.8 (2)
H15E—C15'—H15F	109.5	C55—C54—H54	120.1
C13'—C16'—H16D	109.5	C52—C54—H54	120.1
C13'—C16'—H16E	109.5	C54—C55—C56	119.3 (2)
H16D—C16'—H16E	109.5	C54—C55—H55	120.3
C13'—C16'—H16F	109.5	C56—C55—H55	120.3
H16D—C16'—H16F	109.5	N8—C56—C55	122.4 (2)
H16E—C16'—H16F	109.5	N8—C56—H56	118.8
N3—C17—C18	122.6 (2)	C55—C56—H56	118.8
			
N3—Zn1—S1—C1	92.65 (8)	N2—C13'—C14'—C15'	177.4 (12)
N4—Zn1—S1—C1	27.14 (12)	C16'—C13'—C14'—C15'	−62 (2)
S3—Zn1—S1—C1	−102.80 (7)	C21—N3—C17—C18	1.8 (3)
S4—Zn1—S1—C1	−177.26 (7)	Zn1—N3—C17—C18	−174.46 (18)
S2—Zn1—S1—C1	−0.12 (7)	N3—C17—C18—C19	0.7 (4)
N3—Zn1—S2—C1	−92.95 (8)	C17—C18—C19—C20	−2.4 (4)
N4—Zn1—S2—C1	−166.99 (8)	C18—C19—C20—C21	1.7 (3)
S3—Zn1—S2—C1	98.21 (7)	C18—C19—C20—C22	−179.7 (2)
S1—Zn1—S2—C1	0.12 (7)	C17—N3—C21—C20	−2.5 (3)
N3—Zn1—S3—C9	38.15 (14)	Zn1—N3—C21—C20	174.12 (16)
N4—Zn1—S3—C9	103.67 (8)	C17—N3—C21—C25	179.38 (19)
S4—Zn1—S3—C9	2.60 (7)	Zn1—N3—C21—C25	−3.9 (2)
S1—Zn1—S3—C9	−98.24 (7)	C19—C20—C21—N3	0.8 (3)
S2—Zn1—S3—C9	−171.94 (7)	C22—C20—C21—N3	−177.8 (2)
N3—Zn1—S4—C9	−169.67 (9)	C19—C20—C21—C25	178.9 (2)
N4—Zn1—S4—C9	−95.55 (9)	C22—C20—C21—C25	0.2 (3)
S3—Zn1—S4—C9	−2.61 (7)	C19—C20—C22—C23	−177.7 (2)
S1—Zn1—S4—C9	96.25 (8)	C21—C20—C22—C23	0.9 (3)
N8—Zn2—S5—C29	−101.08 (8)	C20—C22—C23—C24	−0.9 (3)
N7—Zn2—S5—C29	−42.31 (14)	C22—C23—C24—C26	177.4 (2)
S6—Zn2—S5—C29	1.17 (7)	C22—C23—C24—C25	−0.2 (3)
S7—Zn2—S5—C29	97.27 (7)	C28—N4—C25—C24	−2.7 (3)
S8—Zn2—S5—C29	170.94 (7)	Zn1—N4—C25—C24	−171.81 (16)
N8—Zn2—S6—C29	88.24 (9)	C28—N4—C25—C21	176.34 (18)
N7—Zn2—S6—C29	162.16 (9)	Zn1—N4—C25—C21	7.2 (2)
S7—Zn2—S6—C29	−104.12 (7)	C26—C24—C25—N4	2.5 (3)
S5—Zn2—S6—C29	−1.17 (7)	C23—C24—C25—N4	−179.68 (19)
S8—Zn2—S6—C29	−56.09 (13)	C26—C24—C25—C21	−176.47 (19)
N8—Zn2—S7—C37	−40.10 (13)	C23—C24—C25—C21	1.3 (3)
N7—Zn2—S7—C37	−101.43 (9)	N3—C21—C25—N4	−2.2 (3)
S6—Zn2—S7—C37	168.23 (8)	C20—C21—C25—N4	179.62 (19)
S5—Zn2—S7—C37	94.29 (8)	N3—C21—C25—C24	176.82 (19)
S8—Zn2—S7—C37	−2.15 (8)	C20—C21—C25—C24	−1.3 (3)
N8—Zn2—S8—C37	166.44 (9)	C25—C24—C26—C27	−0.6 (3)
N7—Zn2—S8—C37	92.78 (9)	C23—C24—C26—C27	−178.2 (2)
S6—Zn2—S8—C37	−48.28 (13)	C24—C26—C27—C28	−1.1 (3)
S7—Zn2—S8—C37	2.15 (8)	C25—N4—C28—C27	0.9 (3)
S5—Zn2—S8—C37	−100.64 (8)	Zn1—N4—C28—C27	168.68 (16)
N4—Zn1—N3—C17	−178.0 (2)	C26—C27—C28—N4	1.0 (3)
S3—Zn1—N3—C17	−108.4 (2)	C33—N5—C29—S6	169.0 (3)
S4—Zn1—N3—C17	−74.75 (18)	C30—N5—C29—S6	−1.4 (3)
S1—Zn1—N3—C17	29.15 (19)	C33'—N5—C29—S6	−179.3 (3)
S2—Zn1—N3—C17	100.55 (18)	C33—N5—C29—S5	−11.2 (4)
N4—Zn1—N3—C21	5.67 (14)	C30—N5—C29—S5	178.36 (15)
S3—Zn1—N3—C21	75.3 (2)	C33'—N5—C29—S5	0.5 (4)
S4—Zn1—N3—C21	108.89 (15)	Zn2—S6—C29—N5	−178.39 (18)
S1—Zn1—N3—C21	−147.20 (14)	Zn2—S6—C29—S5	1.81 (11)
S2—Zn1—N3—C21	−75.81 (15)	Zn2—S5—C29—N5	178.41 (18)
N3—Zn1—N4—C28	−174.97 (18)	Zn2—S5—C29—S6	−1.80 (11)
S3—Zn1—N4—C28	26.34 (17)	C29—N5—C30—C31	−80.8 (3)
S4—Zn1—N4—C28	99.49 (17)	C33—N5—C30—C31	108.0 (3)
S1—Zn1—N4—C28	−104.76 (18)	C33'—N5—C30—C31	97.1 (4)
S2—Zn1—N4—C28	−78.85 (17)	N5—C30—C31—C32	−179.3 (2)
N3—Zn1—N4—C25	−6.84 (14)	C29—N5—C33—C34	−102.5 (5)
S3—Zn1—N4—C25	−165.54 (13)	C30—N5—C33—C34	68.3 (5)
S4—Zn1—N4—C25	−92.39 (14)	C33'—N5—C33—C34	−179 (3)
S1—Zn1—N4—C25	63.36 (18)	C29—N5—C33—C36	132.6 (4)
S2—Zn1—N4—C25	89.27 (14)	C30—N5—C33—C36	−56.6 (5)
N8—Zn2—N7—C45	179.7 (2)	C33'—N5—C33—C36	56 (3)
S6—Zn2—N7—C45	76.44 (19)	N5—C33—C34—C35	167.9 (6)
S7—Zn2—N7—C45	−23.92 (19)	C36—C33—C34—C35	−67.9 (8)
S5—Zn2—N7—C45	117.29 (19)	C29—N5—C33'—C34'	−110.0 (6)
S8—Zn2—N7—C45	−95.86 (19)	C33—N5—C33'—C34'	0(3)
N8—Zn2—N7—C49	0.28 (14)	C30—N5—C33'—C34'	72.1 (6)
S6—Zn2—N7—C49	−102.99 (14)	C29—N5—C33'—C36'	123.3 (6)
S7—Zn2—N7—C49	156.65 (14)	C33—N5—C33'—C36'	−127 (3)
S5—Zn2—N7—C49	−62.1 (2)	C30—N5—C33'—C36'	−54.6 (7)
S8—Zn2—N7—C49	84.71 (14)	N5—C33'—C34'—C35'	63.2 (10)
N7—Zn2—N8—C56	175.97 (18)	C36'—C33'—C34'—C35'	−167.9 (8)
S6—Zn2—N8—C56	−97.99 (16)	C41—N6—C37—S7	164.3 (3)
S7—Zn2—N8—C56	110.59 (17)	C38—N6—C37—S7	−2.0 (4)
S5—Zn2—N8—C56	−25.76 (17)	C41'—N6—C37—S7	−165.0 (3)
S8—Zn2—N8—C56	74.86 (17)	C41—N6—C37—S8	−13.9 (4)
N7—Zn2—N8—C53	0.78 (14)	C38—N6—C37—S8	179.8 (2)
S6—Zn2—N8—C53	86.82 (14)	C41'—N6—C37—S8	16.7 (4)
S7—Zn2—N8—C53	−64.60 (19)	Zn2—S7—C37—N6	−174.9 (2)
S5—Zn2—N8—C53	159.05 (13)	Zn2—S7—C37—S8	3.36 (12)
S8—Zn2—N8—C53	−100.33 (14)	Zn2—S8—C37—N6	174.9 (2)
C2—N1—C1—S1	1.0 (3)	Zn2—S8—C37—S7	−3.34 (12)
C5—N1—C1—S1	−166.3 (3)	C37—N6—C38—C39	−82.8 (3)
C5'—N1—C1—S1	167.0 (3)	C41—N6—C38—C39	109.7 (3)
C2—N1—C1—S2	179.54 (16)	C41'—N6—C38—C39	79.6 (4)
C5—N1—C1—S2	12.3 (4)	N6—C38—C39—C40	176.0 (3)
C5'—N1—C1—S2	−14.5 (4)	C37—N6—C41—C42	121.0 (4)
Zn1—S1—C1—N1	178.84 (18)	C38—N6—C41—C42	−72.0 (4)
Zn1—S1—C1—S2	0.19 (11)	C41'—N6—C41—C42	37.9 (8)
Zn1—S2—C1—N1	−178.83 (18)	C37—N6—C41—C44	−114.2 (5)
Zn1—S2—C1—S1	−0.19 (11)	C38—N6—C41—C44	52.8 (6)
C1—N1—C2—C3	81.1 (3)	C41'—N6—C41—C44	162.6 (13)
C5—N1—C2—C3	−111.3 (3)	N6—C41—C42—C43	−55.0 (7)
C5'—N1—C2—C3	−85.6 (3)	C44—C41—C42—C43	179.5 (8)
N1—C2—C3—C4	−173.8 (2)	C37—N6—C41'—C42'	−148.7 (4)
C1—N1—C5—C8	108.7 (6)	C41—N6—C41'—C42'	−36.1 (7)
C2—N1—C5—C8	−58.9 (6)	C38—N6—C41'—C42'	48.3 (6)
C5'—N1—C5—C8	−152.3 (17)	C37—N6—C41'—C44'	77.5 (9)
C1—N1—C5—C6	−125.7 (4)	C41—N6—C41'—C44'	−169.8 (15)
C2—N1—C5—C6	66.7 (5)	C38—N6—C41'—C44'	−85.4 (8)
C5'—N1—C5—C6	−26.7 (11)	N6—C41'—C42'—C43'	55.8 (10)
N1—C5—C6—C7	62.5 (8)	C44'—C41'—C42'—C43'	−164.2 (11)
C8—C5—C6—C7	−171.1 (8)	C49—N7—C45—C46	−0.2 (3)
C1—N1—C5'—C6'	132.0 (5)	Zn2—N7—C45—C46	−179.59 (19)
C2—N1—C5'—C6'	−61.6 (6)	N7—C45—C46—C47	−0.4 (4)
C5—N1—C5'—C6'	36.9 (11)	C45—C46—C47—C48	0.7 (4)
C1—N1—C5'—C8'	−94.0 (8)	C46—C47—C48—C49	−0.6 (3)
C2—N1—C5'—C8'	72.4 (8)	C46—C47—C48—C50	−178.8 (2)
C5—N1—C5'—C8'	170.9 (19)	C45—N7—C49—C48	0.3 (3)
N1—C5'—C6'—C7'	−43.3 (10)	Zn2—N7—C49—C48	179.80 (16)
C8'—C5'—C6'—C7'	179.0 (10)	C45—N7—C49—C53	179.3 (2)
C10—N2—C9—S4	−5.1 (3)	Zn2—N7—C49—C53	−1.3 (2)
C13—N2—C9—S4	−176.3 (2)	C47—C48—C49—N7	0.1 (3)
C13'—N2—C9—S4	171.9 (5)	C50—C48—C49—N7	178.4 (2)
C10—N2—C9—S3	173.76 (16)	C47—C48—C49—C53	−178.9 (2)
C13—N2—C9—S3	2.6 (3)	C50—C48—C49—C53	−0.6 (3)
C13'—N2—C9—S3	−9.2 (6)	C49—C48—C50—C51	−0.8 (3)
Zn1—S4—C9—N2	−177.02 (18)	C47—C48—C50—C51	177.4 (2)
Zn1—S4—C9—S3	4.03 (11)	C48—C50—C51—C52	0.7 (4)
Zn1—S3—C9—N2	177.02 (18)	C50—C51—C52—C54	−178.1 (2)
Zn1—S3—C9—S4	−4.03 (11)	C50—C51—C52—C53	0.6 (3)
C9—N2—C10—C11	83.3 (3)	C56—N8—C53—C52	1.8 (3)
C13—N2—C10—C11	−105.2 (3)	Zn2—N8—C53—C52	177.33 (16)
C13'—N2—C10—C11	−93.9 (6)	C56—N8—C53—C49	−177.27 (18)
N2—C10—C11—C12	−176.9 (2)	Zn2—N8—C53—C49	−1.7 (2)
C9—N2—C13—C14	83.7 (4)	C54—C52—C53—N8	−2.1 (3)
C10—N2—C13—C14	−87.7 (4)	C51—C52—C53—N8	179.05 (19)
C13'—N2—C13—C14	170 (4)	C54—C52—C53—C49	176.87 (19)
C9—N2—C13—C16	−142.2 (3)	C51—C52—C53—C49	−1.9 (3)
C10—N2—C13—C16	46.4 (4)	N7—C49—C53—N8	2.0 (3)
C13'—N2—C13—C16	−56 (3)	C48—C49—C53—N8	−179.02 (19)
N2—C13—C14—C15	68.7 (5)	N7—C49—C53—C52	−177.07 (19)
C16—C13—C14—C15	−65.1 (5)	C48—C49—C53—C52	1.9 (3)
C9—N2—C13'—C14'	−130.7 (9)	C53—C52—C54—C55	0.6 (3)
C10—N2—C13'—C14'	46.4 (12)	C51—C52—C54—C55	179.3 (2)
C13—N2—C13'—C14'	130 (4)	C52—C54—C55—C56	1.3 (3)
C9—N2—C13'—C16'	102.9 (10)	C53—N8—C56—C55	0.2 (3)
C10—N2—C13'—C16'	−80.0 (11)	Zn2—N8—C56—C55	−174.88 (16)
C13—N2—C13'—C16'	3(3)	C54—C55—C56—N8	−1.7 (3)

### Hydrogen-bond geometry (Å, °)

**Table:**

*D*—H···*A*	*D*—H	H···*A*	*D*···*A*	*D*—H···*A*
C28—H28a···S8^i^	0.95	2.80	3.560 (2)	137
C50—H50···S8^ii^	0.95	2.80	3.702 (3)	158

Symmetry codes: (i) *x*−1, *y*, *z*; (ii) −*x*+2, −*y*+1, −*z*+1.
